# Simple Treatment of Corns

**Published:** 1911-11

**Authors:** 


### Simple Treatment of Corns.

Griffith of New York, in the September *Medical Review*, says: "A strip of adhesive plaster from three-eighths to one-half of an inch (1 cm.-1-25 cm.) in width and four to six (10 cm.-15 cm.) inches long is to be applied in spiral fashion to the affected toe, covering the digit from neck to nail. The degree of tightness of the application deserves consideration to avoid compression. However, the feelings of the patient when stepping upon the foot will serve as an adequate guide in this matter. Given instructions to cut through the plaster lengthwise or to soak off the entire dressing by immersion in a hot-water foot bath, affords ample protection in cases of undetected microbic infection. Soaking the foot for ten to twenty minutes in water at a temperature of 100° F., with gentle removal by rubbing with a piece of sterilized pumice stone or forceps of the crown of hardened epidermis shortens the time of treatment. Properly applied, the plaster strap dressing described should afford relief from the moment of its application, and may be worn continuously for from one to six or eight weeks—bathing seeming to unaffect the adhesive properties of the plaster after having once set. Removal of the dressing at the end of an adequate time reveals the cornus completely freed, when it may be picked out entire by means of a dressing forceps, or after an additional soaking. A wisp of absorbent cotton, held on by means of a narrow adhesive strap, may be subsequently worn for a few days."—*Lancet-Clinic.*

